# Commentary: Outcome Predictors of Biopsy-Proven Myeloperoxidase-Anti-Neutrophil Cytoplasmic Antibody-Associated Glomerulonephritis

**DOI:** 10.3389/fimmu.2021.691179

**Published:** 2021-06-02

**Authors:** Samuel Wacrenier, Charlotte Boud’hors, Giorgina Piccoli, Jean-François Augusto, Benoit Brilland

**Affiliations:** ^1^ Service de Néphrologie-Dialyse-Transplantation, Université d’Angers, CHU Angers, Angers, France; ^2^ Service de Néphrologie, Centre Hospitalier du Mans, Le Mans, France; ^3^ Université d’Angers, INSERM, CRCINA, Angers, France

**Keywords:** ANCA-associated vasculitis, glomerulonephritis, predictor, death, end stage kidney disease

## Introduction

Diagnosis of Antineutrophil cytoplasmic antibodies (ANCA) associated vasculitis (AAV) with glomerulonephritis (ANCA-GN) enjoins the start of an immunosuppressive treatment as soon as possible. Its intensity is guided by clinical presentation and histopathologic lesions on kidney biopsy. Individualized and personalized treatment is a key issue for improving outcomes. In this view, markers of AAV severity, renal involvement, and overall prognosis may be useful, not only for a better understanding of the disease pathophysiology, but above all, for guiding therapies and improving patients’ outcomes, especially when histology is not available. Moreover, if chronic lesions in kidney biopsy at diagnosis remain a major determinant of renal outcomes, whether assessed by the international histopathologic classification (1), the Renal Risk Score (2) or the global chronicity score proposed by the Mayo Clinic (3), some intermediate prognostic classes (*e.g.* crescentic and mixed classes in Berden’s classification) actually seem to have similar renal outcomes (4). Thus, biomarkers are needed to refine the prognosis assessment of these patients.

We therefore read we much interest the publication by Ge et al. regarding the role of several biological markers associated with death and end stage kidney disease (ESKD) in MPO-ANCA vasculitis patients with pauci-immune glomerulonephritis (MPO-ANCA-GN), especially anemia and hypoalbuminemia (5). In their cohort of 112 patients, albuminemia < 30 g/L and hemoglobin < 9g/dL were associated with a greater risk of ESKD occurrence. Moreover, hypoalbuminemia (but not anemia) was associated with patient survival.

## Predictors of Death and ESKD in the Maine-Anjou Registry

We aimed to evaluate the predictive value of these biological markers within our cohort of AAV patients with biopsy-proven kidney involvement, included in the Maine-Anjou (France) registry. This population has previously been described in various settings (6–9). Among the 180 patients within the registry, 111 patients presented with MPO-ANCA of whom 98 had ANCA-GN histological confirmation with kidney biopsy. To meet the same inclusion criteria used by Ge et al., we analyzed these 98 patients. Median age was 68 years old [61-75], 61% were male and more than half had hypertension. Median BVAS was 14 [12-18], and, after kidneys, most involved organs were lungs (33%), ear, nose and throat (22%) and skin (13%). At baseline and before any therapeutic intervention, median eGFR was 17 ml/min [12, 33], median hemoglobin was 10 g/dL [8.75 - 10.95] of whom 31% had a hemoglobin level < 9g/dL and median albuminemia was 30 g/L [28 - 34] of whom 45% had an albumin level < 30 g/L. A fifth (21%) required kidney replacement therapy within 30 days from diagnosis. According to Berden’s classification, the proportion of patients within the focal class, the crescentic class, the mixed class and the sclerotic class was 19%, 43%, 20% and 18%, respectively. In addition to oral glucocorticoids, most patients received intravenous methylprednisolone pulses (81%) associated with cyclophosphamide (84%) or rituximab (16%) as induction therapy. Maintenance therapy mostly consisted in azathioprine or rituximab regimens (57% and 41%, respectively). These 98 patients were followed with a median follow-up of 46 months [16-96] during which 25 patients (26%) experienced a relapse (60% with renal involvement), 28 patients (29%) reached ESKD and 24 patients (24%) died.

In our cohort, among clinical or biological parameters at diagnosis, albuminemia (either considered as a continuous variable or as a dichotomized one) was neither associated with death nor with ESKD (5-years censured Multivariable Cox Analysis). Among the different models used, age and proteinuria were the best predictors for death occurrence. The predictive value of anemia appeared to be inconsistent (**Table 1**. The best predictor for ESKD occurrence was the severity of renal involvement at admission (amount of proteinuria and the need for kidney replacement therapy within 30 days from diagnosis). Similarly to albuminemia, hemoglobin (either considered as a continuous variable or dichotomized at anemia threshold) was not associated with ESKD (**Table 1**).

**Table 1:** 5-years censured Cox Multivariable analysis of patient survival (death) or renal survival (ESKD).

	Multivariable analysis of death predictors							Multivariable analysis of ESKD predictors					
Model 1: including albumin and hemoglobin as continuous variables													
	Full model			Simplified model **				Full model			Simplified model **		
	HR	95% CI	p-value	HR	95% CI	p-value		HR	95% CI	p-value	HR	95% CI	p-value
Age (per 10 years increment)	2.34	1.02, 5.37	**0.045**	2.13	1.03, 4.40	**0.041**	Creatinine (per 50 µmol/L increment)	1.01	0.90, 1.12	>0.9			
Hypertension	8.47	0.80, 90.0	0.076	10.8	1.08, 107	**0.043**	Proteinuria (per 1 g/g increment)	1.06	0.94, 1.20	0.3	1.08	1.02, 1.14	**0.014**
Creatinine (per 50 µmol/L increment)	0.99	0.87, 1.11	0.8				Hemoglobin (per 1 g/dL increment)	0.92	0.72, 1.17	0.5			
Proteinuria (per 1 g/g increment)	1.19	1.02, 1.38	**0.026**	1.17	1.06, 1.29	**0.001**	Albumin (per 1 g/L increment) *	1.01	0.94, 1.09	0.8			
Hemoglobin (per 1 g/dL increment)	0.65	0.43, 0.97	**0.034**	0.63	0.44, 0.90	**0.011**	KRT within 30 days from diagnosis	8.06	2.33, 27.9	**<0.001**	8.09	3.43, 19.1	**<0.001**
Albumin (per 1 g/L increment) *	1.01	0.91, 1.11	>0.9										
Model 2: including albumin and hemoglobin as dichotomous variables: thresholds from Ge et al.													
	Full model			Simplified model **				Full model			Simplified model **		
	HR	95% CI	p-value	HR	95% CI	p-value		HR	95% CI	p-value	HR	95% CI	p-value
Age (per 10 years increment)	2.60	1.12, 6.01	**0.026**	3.60	1.86, 6.96	**<0.001**	Creatinine (per 50 µmol/L increment)	1.01	0.91, 1.13	0.8			
Hypertension	6.06	0.66, 55.3	0.11				Proteinuria (per 1 g/g increment)	1.05	0.94, 1.18	0.4	1.08	1.02, 1.14	**0.014**
Creatinine (per 50 µmol/L increment)	0.93	0.79, 1.09	0.4				Hemoglobin (< 9 g/dL) *	1.17	0.44, 3.08	0.8			
Proteinuria (per 1 g/g increment)	1.27	1.05, 1.54	**0.012**	1.21	1.09, 1.34	**<0.001**	Albumin (< 30 g/L) *	0.72	0.28, 1.83	0.5			
Hemoglobin (< 9 g/dL) *	1.72	0.51, 5.82	0.4				KRT within 30 days from diagnosis	8.86	2.56, 30.6	**<0.001**	8.09	3.43, 19.1	**<0.001**
Albumin (< 30 g/L) *	0.67	0.19, 2.38	0.5										

Cox proportional hazards regression analysis was performed to examine factors associated with the occurrence of death and ESKD. Multivariate Cox regression analysis included all parameters with p < 0.1 in the univariate analysis or parameters judged as clinically relevant (*). Simplified models were built using manual step-by-step backward selection with a removal criterion of p > 0.1 (**).

KRT, kidney replacement therapy; HR, hazard ratio; CI, confidence interval. p-values < 0.05 are in bold.

## Discussion

Our results underline the inconsistent predictive value of these biological markers (hemoglobin and albumin) when applied to our cohort. Indeed, as stated by Ge et al., literature is very heterogeneous on this subject. Hemoglobin has been found irregularly as a predictor of death (10–12) or not (13) and as a predictor of ESKD (11) or not (14, 15). In the same way, albumin has been found as a predictor of death (16) or not (17, 18) and as a predictor of ESKD (19) or not (20, 21). These inconsistent results may be explained by various collinear variables that can unspecifically and falsely bring out biological predictors. For example, the amount and duration of inflammatory syndrome, the nutritional status before ANCA-GN onset, and the severity (and chronicity) of kidney failure can all influence these parameters. Moreover, these studies vary in term of number of patients, ANCA type, severity at presentation and management. While we included a similar number of patients with biopsy-proven MPO-ANCA-GN, approximatively followed for the same duration as compared to Ge et al. study, the discrepancy between the two cohorts may be explained by additional factors. First, the Asian population experienced more severe outcomes (death and ESKD rate reached 41% and 39%, respectively) possibly related to a more severe initial clinical presentation (median BVAS 18 and need for KRT 42%). Moreover, remission induction regimen was based on glucocorticoids and mycophenolate mofetil (MMF) for a non-negligible proportion of patients (20.5%). This is not surprising as MMF-based induction regimen was associated with better outcomes in Chinese patients when compared with conventional European immunosuppressant induction therapy such as cyclophosphamide (22). Maintenance regimen was also different as MMF and cyclophosphamide was predominantly used in Ge et al. cohort, whereas we mostly used azathioprine and rituximab. Thus, ethnicity, a more severe initial presentation and different therapeutic management may explain these differences.

To conclude, in the quest of the perfect biological marker, we would like to bring out that hemoglobin and albumin may not be the eagerly awaited ones. Beyond the classical aphorism “the worse it is, the worse it gets”, various biomarkers are currently under careful examination to reach this goal (23). Some may seem duller than others but nevertheless just as promising. Indeed, we recently reported lymphopenia as a strong independent predictor of ESKD (9) and think that exploring abnormalities in lymphocytes phenotype, whether considering kidney infiltrating cells or circulating lymphocytes, would be enlightening in this setting. Indeed, lymphopenia by itself (9) but also the underlying mechanisms (*e.g.* loss of regulatory cells, regulatory/inflammatory cells imbalance, and, activated T cells infiltration) (24–27) could be of great value to help predict bad outcomes in ANCA-GN.

## Author Contributions

SW, BB, and JFA designed the study. SW, CB, and BB gathered the information included in the database. SW and BB analyzed the data. SW wrote the first draft of the manuscript. BB and J-FA revised the manuscript. All authors participated in patient care. All authors contributed to the article and approved the submitted version.

## Conflict of Interest

The authors declare that the research was conducted in the absence of any commercial or financial relationships that could be construed as a potential conflict of interest.
